# Creating, Recruiting, and Developing: Key Tasks and Their Challenges

**DOI:** 10.1093/geroni/igab046.1676

**Published:** 2021-12-17

**Authors:** Edward Ansello, Faith Helm

**Affiliations:** 1 Virginia Commonwealth University, Richmond, Virginia, United States; 2 University of Rhode Island, Kingston, Rhode Island, United States

## Abstract

The ECHO model is uniquely suited to developing education for a wide range of agencies and providers serving the needs of older adults with IDD. The program’s structure and its educational philosophy depend on modeling teamwork in both the hub and the spokes. Recruitment of participants included paid caregivers, healthcare practitioners, and direct service providers, focusing on team participation at each site. In developing the curriculum, it was critical to recognize the roles played by each sector, as well as the complementary contributions of others. Consequently, curriculum content needed to be multidisciplinary and multifocal, and recognize both the breadth of contributors and time limits in selecting content for each session. Didactic presentations and case studies embodied these features. Priorities included best practices in person-centered care; differential diagnoses; and physical, social, and environmental factors. The facilitators of, and challenges to, these priorities offer implications for advancing educational programs with similar objectives.

